# Selectivity of Direct Methanol Fuel Cell Membranes

**DOI:** 10.3390/membranes5040793

**Published:** 2015-11-24

**Authors:** Antonino S. Aricò, David Sebastian, Michael Schuster, Bernd Bauer, Claudia D’Urso, Francesco Lufrano, Vincenzo Baglio

**Affiliations:** 1 CNR-ITAE Institute for Advanced Energy Technologies “N. Giordano”, Via Salita S. Lucia sopra Contesse 5, Messina 98126, Italy; E-Mails: sebastian@itae.cnr.it (D.S.); durso@itae.cnr.it (C.D.U.); lufrano@itae.cnr.it (F.L.); baglio@itae.cnr.it (V.B.); 2 FuMA-Tech Gesellschaft für funktionelle Membranen und Anlagentechnologie mbH, Carl-Benz-Strasse 4, Bietigheim-Bissingen D-74321, Germany; E-Mails: schuster@fumatech.de (M.S.); bernd.bauer@fumatech.com (B.B.)

**Keywords:** proton exchange polymer electrolyte membranes, direct methanol fuel cells, methanol crossover, proton conductivity, membrane selectivity, fuel cell performance

## Abstract

Sulfonic acid-functionalized polymer electrolyte membranes alternative to Nafion^®^ were developed. These were hydrocarbon systems, such as blend sulfonated polyetheretherketone (s-PEEK), new generation perfluorosulfonic acid (PFSA) systems, and composite zirconium phosphate–PFSA polymers. The membranes varied in terms of composition, equivalent weight, thickness, and filler and were investigated with regard to their methanol permeation characteristics and proton conductivity for application in direct methanol fuel cells. The behavior of the membrane electrode assemblies (MEA) was investigated in fuel cell with the aim to individuate a correlation between membrane characteristics and their performance in a direct methanol fuel cell (DMFC). The power density of the DMFC at 60 °C increased according to a square root-like function of the membrane selectivity. This was defined as the reciprocal of the product between area specific resistance and crossover. The power density achieved at 60 °C for the most promising s-PEEK-based membrane-electrode assembly (MEA) was higher than the benchmark Nafion^®^ 115-based MEA (77 mW·cm^−2^ *vs.* 64 mW·cm^−2^). This result was due to a lower methanol crossover (47 mA·cm^−2^ equivalent current density for s-PEEK *vs.* 120 mA·cm^−2^ for Nafion^®^ 115 at 60 °C as recorded at OCV with 2 M methanol) and a suitable area specific resistance (0.15 Ohm cm^2^ for s-PEEK *vs.* 0.22 Ohm cm^2^ for Nafion^®^ 115).

## 1. Introduction

Direct methanol fuel cells (DMFCs) based on solid protonic electrolytes and operating at low temperatures (up to 90 °C) are widely considered for application in portable power sources, remote energy generation, and auxiliary power units (APU) in both stationary and mobile applications [1,2]. The specific advantages of such systems over the competing technologies are related to their simplicity of operation, high energy density, lightweight, compactness, and fast refilling properties. DMFCs utilize methanol as liquid fuel to deliver continuous power; in this regard, they have fewer constraints than hydrogen-fueled fuel cells in terms of fuel handling, refueling, and fuel storage processes. However, there are still specific drawbacks for DMFCs in terms of high cost and low performance, and the stability needs to be improved [2,3]. The main objective of the research in the field of direct methanol fuel cells [3,4,5,6,7] is to develop cost-effective catalysts and membranes with enhanced properties in order to reduce stack costs and improve performance and durability. Concerning the membrane, the main purpose of the developmental activities is to provide enhanced conductivity at a reduced methanol crossover [8]. In principle, the conductivity of the state-of-the-art Nafion^®^ membrane, which is in the range of 0.1 S·cm^−1^, appears very appropriate for DMFC devices but the methanol permeability for this polymer electrolyte is unfortunately also significant, especially under open circuit conditions [3,9].

Both methanol crossover and conductivity increase in hydrophilic membranes with the membrane hydration level (λ). Thus, an enhancement of one of these properties will have a significant impact on the other. Methanol has high interaction with polymer chains and high solubility in water, and its permeation easily occurs through the hydrophilic water channels inside the membrane. A network of widely distributed and interconnected ionic domains that, under proper hydration, give rise to water channels, is essential to achieve a high proton conduction in these membranes. Perfluorinated membranes containing sulfonic acid functionalities, such as Nafion^®^, are, at present, the best performing systems at temperatures lower than 90 °C. This is due to a proper combination of proton conductivity, mechanical and hydrolytic stability, as well as good interface properties. The sulfonic acid groups are characterized by a high level of dissociation in the presence of suitable water contents. Membrane ionic conductivity is essentially promoted by proton diffusion through the vehicle mechanism [10,11,12,13]; according to this mechanism, protons are transported by water molecules.

Reduction of methanol crossover can be achieved by using cross-linking procedures or adding nanosized inorganic fillers inside the membrane to increase the tortuosity path as well as by tuning the ion exchange capacity (IEC) [2,14].

Another approach considers the variation of the chemical properties of the polymer network surrounding the ionic groups to modulate the degree of dissociation as well as the degree of interpenetrated networks [15,16,17].

These approaches can reduce the level of methanol permeation permeability while keeping the proton conductivity at suitable levels. This allows us to achieve both convenient fuel utilization and appropriate power density since the lower the methanol permeation the smaller are the effects related to the occurrence of a mixed potential at the cathode [1]. Accordingly, the cathode is less polarized in the presence of lower methanol permeation. This corresponds to lower overpotentials for the oxygen reduction reaction [18,19]. This strategy is further assisted by using methanol-tolerant cathode electro-catalysts with high activity for oxygen reduction [18,19].

It is considered that the occurrence of ionic conductivity better than 50 mS·cm^−1^ and methanol crossover lower than 5 × 10^−6^ mol·cm^−2^·min^−1^ for a membrane with a thickness lower than 100 µm in the presence of low overpotentials for anode/cathode reactions (<0.3 V at 100 mA·cm^−2^) may result in an appropriate performance (>50 mW·cm^−2^ at 60 °C) in the presence of moderate catalyst loading (1–2 mg·cm^−2^). A suitable level of power density together with a proper fuel utilization are the main pre-requisites for fuel cells to compete with batteries in portable power systems and to find application as alternative power generators in auxiliary power units. In this regard, a more in-depth understanding of the influence of membrane properties on the performance of DMFCs is necessary.

The present work was addressed to study the effect of varying membranes properties such as methanol permeability, thickness, and proton conduction on the behavior of direct methanol fuel cells. The investigated membranes were essentially perfluorinated systems and aromatic hydrocarbon polymers, such as polyetheretherketone (PEEK), functionalized with sulfonic acid groups. These polymer electrolytes were varied in terms of equivalent weight, composition, filler content, thickness, cross-linking to modulate the proton conductivity, and methanol permeability with the aim to investigate their influence on the DMFC performance.

## 2. Results and Discussion

### 2.1. Membrane Characteristics

Proton exchange membranes based on hydrocarbon and perfluorosulfonic acid-type polymers were developed. Various compositions (nature of polymer backbone, presence or absence of an inorganic component or cross-linking), polymer equivalent weight (EW), and membrane thickness have been explored. FuMA-Tech developed a range of low IEC (high EW) membranes designed for low methanol crossover that were based upon long side chain (Fumion) PFSA blends [20,21]. The nominal EWs of these membranes were 1400, 1800, and 2300 g/mol. Another series of FuMA-Tech PFSA membranes incorporated a stabilizer (labeled as FX) to mitigate radical attack. FuMA-Tech also prepared blend membranes of various thicknesses based upon sulfonated polyetheretherketone, with only 30 µm thickness in one case (E-730) [12,22]. Another approach at FuMA-Tech was dealing with composite membranes based on a PFSA polymer where zirconium phosphate nanoparticles were integrated into the PFSA polymer matrix. These composite membranes based on zirconium phosphate (ZrP = Zr(HPO_4_)_2_·H_2_O) and perfluorosulfonic acid polymer (PFSA) are labeled as fumapem^®^ FZP. Inclusion of ZrP into a PFSA matrix is known to increase mechanical stability at elevated temperatures [23,24]. Furthermore, the presence of layered ZrP nanoparticles in the PFSA polymer matrix may also reduce methanol crossover [23,25]. This approach allowed the continuous production of composite membranes using the standard production procedure for membranes at FuMA-Tech where the ZrP nanoparticles were formed *in situ* during the casting process.

The main properties of the most promising membranes in comparison to Nafion 115 are discussed in detail in the following. Among the long side chain (Fumion), PFSA blends with nominal EWs of 1400, 1800, and 2300, the F-1850 (EW = 1800) membrane provided promising conductivity values of 47 mS/cm at 60 °C and 84 mS/cm at 100 °C (measured at 95% RH).

The high-EW PFSA membranes named fumapem^®^ F-1850 (FuMA-Tech) had an EW = 1800 g/mol and a thickness of 50 µm. This was selected as the best compromise between area resistance, methanol permeation, fuel cell performance, mechanical strength, processability, handleability, and material cost. A key features of the fumapem^®^ 1850 was a reduced area resistance while maintaining methanol permeability lower than Nafion^®^ 115. This resulted in a better DMFC performance than Nafion^®^ 115 at 60 °C. However, most important is the fact that fumapem^®^ F-1850 has the potential to reduce the cost of materials by a factor of 4 compared to Nafion^®^ 115. FuMA-Tech also prepared blend membranes of various thicknesses based upon sulfonated polyetheretherketone. The E-730 membrane, even at only 30 µm in thickness, had a reasonably low methanol crossover, and therefore good fuel utilization and high power density.

*Ex* *situ* characterization data of fumapem^®^ F-1850 and E-730 membranes are shown in Table 1 in comparison to Nafion^®^ 115. This table also includes the FZP960 and FX7050 membranes to provide a dataset of the properties of the most promising membranes for each composition.

**Table 1:** *Ex situ* characterization data of the most promising Fumatech membranes for each category compared to Nafion^®^ 115.

Membrane Acronym	Unit	F-1850	E-730	FZP-960	FX-7050	N-115
Polymer Type		PFSA	sPEEK	PFSA-ZrP	PFSA Cross-Linked	PFSA
Filler content	–	–	–	10%	–	–
EW (theoretical)	g/mol	1800	740	950	7000	1100
IEC (exp.)	mmol/g	0.50	1.35	0.83	0.63	0.9
Thickness (dry)	µm	50	30	60	50	125
Solvent uptake in MeOH at 25 °C	Wt %	30	38	115	33	54
Length increase Δl in MeOH at 25 °C	%	18	10	46	8	31
Conductivity in H_2_O at T = 25 °C	mS·cm^−1^	58	16	23	56	62

Conductivity, swelling, and methanol uptake varied significantly in these membranes as a consequence of different chemistry and equivalent weight.

Three composite membranes containing ZrP (FZP-960, FZP-990 and FZP-9110) with equivalent weight 950 g/mol and thicknesses of 60, 90, and 110 µm were prepared. *Ex situ* conductivity measurements in water at room temperature of ZrP-PFSA composite membranes (FZP series) showed conductivities between 40 and 70 mS·cm^−1^ after activation, close to standard PFSA membranes. Without activation, all fumapem^®^ FZP membranes showed a conductivity around 10 mS·cm^−1^. The conductivity of FZP9110 was 68 mS·cm^−1^; this favorably compared to that observed for Nafion, 62 mS·cm^−1^, under similar conditions. However, no significant reduction of the swelling behavior in methanol at high concentrations was observed for this series of membranes, rather a slight increase compared to Nafion^®^ 115. An approach was devoted to prepare a PFSA ionomer containing a chemical/mechanical stabilizer, mainly intended to reduce swelling in methanol. Membranes with different amounts of stabilizer were produced; these membranes are labeled as fumapem^®^ FX (stabilized PFSA). Overall, the addition of a stabilizer obviously had a weakening effect on the membrane mechanical properties when treated with methanol solution; however, by increasing the amount of stabilizer, the membrane swelling in methanol was again reduced. Unfortunately, increasing the amount of stabilizer meant that the conductivity decreased. For DMFC testing, fumapem^®^ FX-7050 was selected for this series because it showed the lowest swelling in methanol but still sufficiently high conductivity. The FX-7050 consisted of an equivalent weight of 7000 and had a thickness of 50 µm.

### 2.2. Electrochemical Properties

The membranes were investigated at CNR-ITAE in DMFC, at different temperatures, to get insights into their practical applications. The membranes were characterized in terms of polarization behavior under fuel cell conditions, cell resistance during operation (by ac-impedance spectroscopy), analysis of the equivalent methanol crossover current density, and compared to the benchmark Nafion^®^ 115 benchmark membrane. Catalyst loading was 2 mg PtRu·cm^−2^ at the anode and 1 mg Pt·cm^−2^ at the cathode.

The crossover of methanol was determined using two methods. One method concerned the analysis of the exhaust gas at the cathode side of the DMFC single cell under open circuit voltage (OCV). The CO_2_ produced by the oxidation of the methanol permeated to the cathode was analyzed using an online micro-gas chromatograph; the second approach consisted of a linear sweep voltammetry of the DMFC operating in a driven-mode with the cathode fed with inert gas [26]. In the latter case, methanol permeated to the cathode was completely oxidized at high electrochemical potentials (>0.9 V). Both methods provided consistent results. Methanol concentrations of 1, 2, and 5 M were investigated. In both procedures, sampled aliquots of the condensed cathode stream were analyzed in a bench gas-chromatograph equipped with a flame ionization detector. Only traces of unreacted methanol were detected in the condensed stream from the cathode.

As is well known, methanol crossover decreases with the operating current density [2,3]. In our analysis, we have preferred to measure methanol crossover under OCV since this condition corresponds to the maximum conversion of permeated methanol to CO_2_ at the cathode due to the high electrochemical potential.

**Figure 1:**
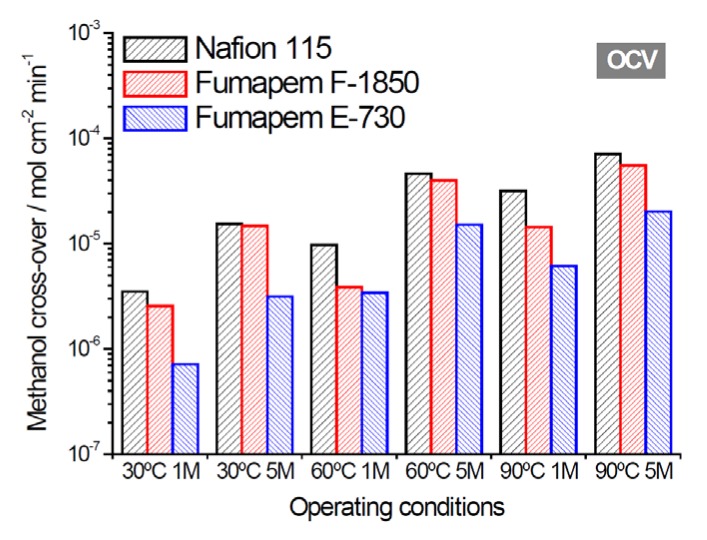
Methanol crossover for selected membranes under different operating conditions of temperature and methanol concentration; crossover was here determined at open circuit voltage (OCV) during normal DMFC operation by measuring the CO_2_ evolved at the cathode using an in-line chromatograph.

Figure 1 provides a comparison of methanol crossover values for some of the investigated membranes and the commercial Nafion^®^ 115 in the presence of 1 and 5 M methanol. This measurement was carried out by analyzing the CO_2_ at the cathode using the online gas chromatograph. The crossover increased in the order E-730 < F-1850 < Nafion^®^-115.

In general, crossover values are affected by methanol uptake, permeability and membrane thickness. However, thick membranes can result in high cell resistances, and this leads to performance losses.

**Figure 2:**
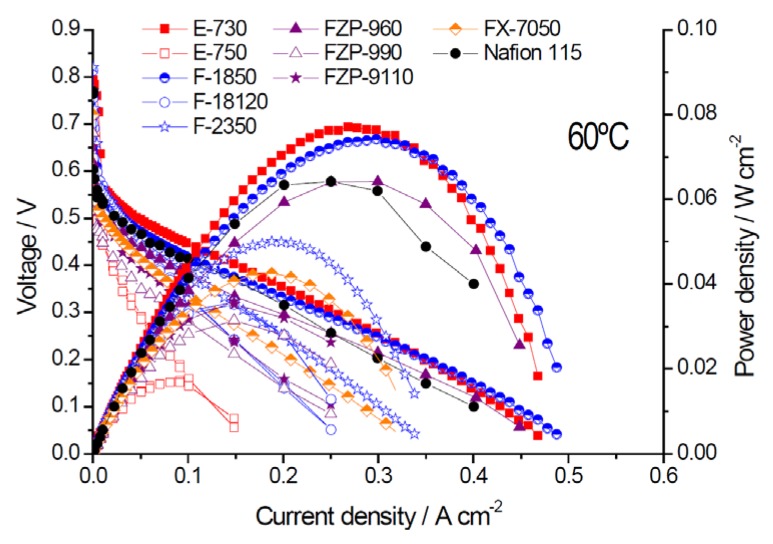
Polarization and power density curves for different membranes in MEAs at 60 °C, 2 M methanol.

**Figure 3:**
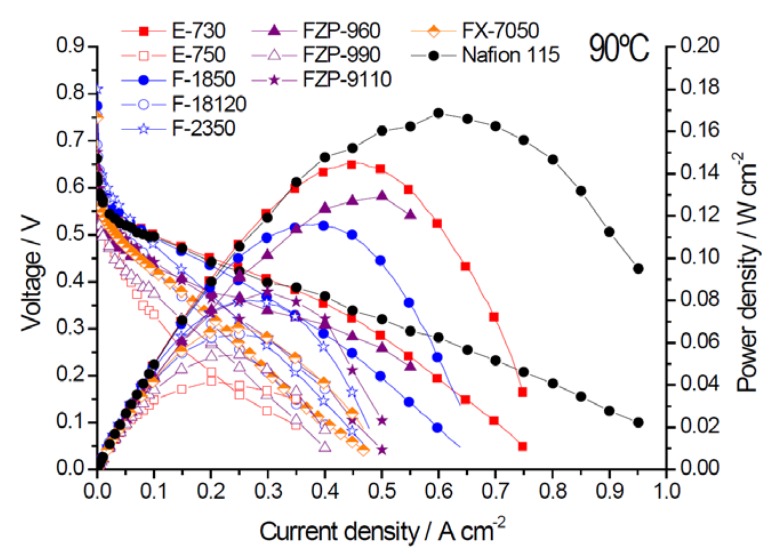
Polarization and power density curves for the different membranes in MEAs at 90 °C, 2 M methanol.

The polarization and power density (P.D.) curves, obtained at 60° and 90 °C for various MEAs based on different membranes, are reported in Figure 2 and Figure 3. From these curves, it appears that the MEA based on s-PEEK (E-730) is the best performing one for operation in the low temperature range. At low temperatures, the performance obtained with the E-730 membrane was higher than that achieved with Nafion^®^ 115 membrane and similar to the fumapem^®^ F-1850.

The main results in terms of performance, crossover, and area-specific resistance recorded at 60 °C and 90 °C are summarized in Table 2. All data in Table 2 are for DMFC operation with 2 M methanol and the reported crossover measurements were carried out in this case by linear sweep voltammetry with the DMFC cell operating in the driven mode.

The E-730 membrane showed better performance than Nafion^®^ at 60 °C (77 *vs.* 64 mW·cm^−2^) as a result of the much lower methanol crossover (48 *vs.* 110 mA·cm^−2^ equivalent current density). The thin F-1850 membrane, characterized by an equivalent weight of 1800, performed much better than the F-18120 characterized by same EW but had a larger thickness (50 µm *vs.* 120 µm). The membrane with similar chemistry e.g., the F-2350 with 50 µm thickness, but having larger EW, *i.e.*, 2300, exhibited lower performance. If we compare at 60 °C the power density of the most promising PFSA membrane, *i.e.*, F-1850, to the Nafion^®^ 115 benchmark, it clearly appears that a performance increase of about 15% is achieved for the FUMATECH membrane. This enhanced performance is essentially due to the lower methanol crossover. At 60 °C, methanol crossover for the F-1850 was less than half that of Nafion^®^ despite the fact that the F-1850 membrane was much thinner. The maximum power densities of 74 and 77 mW·cm^−2^ at 60 °C for the F-1850 and E-730 were quite promising considering the reasonably low catalyst loading.

At 90 °C, Nafion^®^ 115 performed better than the other membranes in terms of peak power density. This can be interpreted by the fact that at high temperatures, the poisoning effect caused on the cathode by methanol crossover is less dramatic since at 90 °C methanol species are not strongly adsorbed on the electrode surface [27]. This provides a better capability to the O_2_ molecules to compete for adsorption on the cathode surface. Under these conditions, the excellent interfacial properties of Nafion^®^ play a significant role. However, the effect of crossover is still relevant for Nafion^®^, especially at low current densities.

**Table 2:** Electrochemical characteristics of the membranes in DMFC (2M CH_3_OH).

Membrane Acronym	Units	E-730	E-750	F-1850	F-18120	F-2350	FZP-960	FZP-990	FZP-9110	FX-7050	Nafion^®^ 115
Polymer Type		s-PEEK	s-PEEK	PFSA	PFSA	PFSA	PFSA-ZrP	PFSA-ZrP	PFSA-ZrP	PFSA Cross-Linked	PFSA
Equivalent weight	g/mol	700	700	1800	1800	2300	950	950	950	7000	1100
thickness	µm	30	50	50	120	50	60	90	110	50	125
Max. Power density	mW·cm^−2^	77	17	74	36	50	64	32	35	42	64
	@ 60 °C										
R_s_ (EIS)	Ω cm^2^	0.20	0.62	0.20	0.50	0.31	0.085	0.29	0.33	0.13	0.17
	@ 60 °C										
Crossover current	mA·cm^−2^	48	106	120	100	88	128	135	208	186	195
	@ 60 °C										
Max. Power density	mW·cm^−2^	145	42	116	64	80	129	55	84	67	167
	@ 90 °C										
R_s_ (EIS)	Ω cm^2^	0.14	0.23	0.16	0.38	0.34	0.06	0.21	0.24	0.12	0.14
	@ 90 °C										
Crossover current	mA·cm^−2^	74	185	193	123	121	153	162	245	260	380
	@ 90 °C										

From this survey, it appears that thin membranes, both bare and composite ones, are better performing than the thicker ones in the same series. The membrane based on s-PEEK, 30 µm in thickness (E-730), provides higher performance and more moderate methanol crossover than the membrane based on the same polymer with 50 µm in thickness (E-750) characterized by a larger cell resistance. The same behavior was recorded with the 50-µm membrane based on PSFA (F-1850) compared to the thicker F-18120 (120 µm). The three composite membranes containing Zr-phosphate appeared less promising, similarly to the cross-linked FX-7050 membrane containing the stabilizer.

The membrane based on s-PEEK (E-730) was the best performing one for operation in the low temperature range. At 60 °C, the performance for the E-730 membrane was better than that obtained with Nafion^®^ 115 membrane and slightly better than the fumapem^®^ F-1850 (see Table 2).

### 2.3. Membrane Selectivity

To better understand the combined effect of membrane conductivity, thickness, and methanol permeation, we have here calculated the membrane selectivity, s, as the reciprocal of the product of the area-specific resistance (series resistance), R_s_, multiplied by the crossover equivalent current density, I_crossover_:

s = 1/(R_s_·I_crooss-over_), (1)

where s ≡ S·mA^−1^; R_s_ ≡ Ohm cm^2^ and I_crossover_ ≡ mA·cm^−2^; S ≡ Siemens.

The area-specific resistance or series resistance was measured *in situ* using ac-impedance spectroscopy; this parameter includes both the contributions of membrane conductivity and thickness:

R_s_ = t/σ, (2)

where R_s_ is the series resistance (ohm cm^2^); t is the thickness (cm) and σ is the conductivity (ohm^−1^·cm^−1^ or S·cm^−1^).

**Figure 4:**
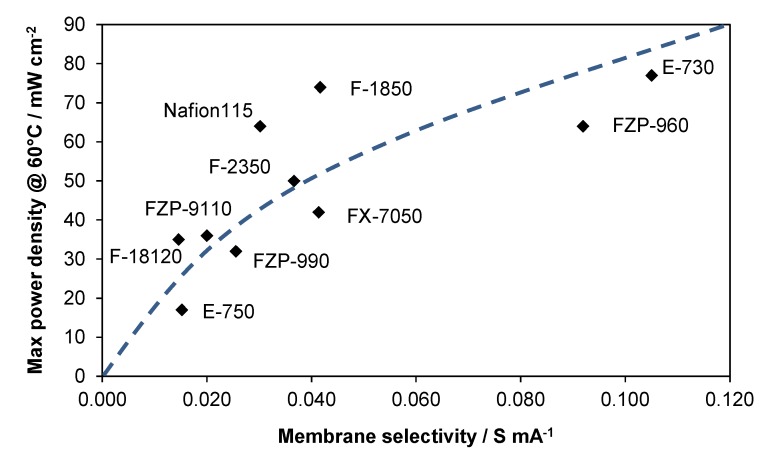
Performance *vs.* selectivity for MEAs equipped with different membranes at 60 °C (2 M MeOH).

By analyzing the data obtained at 60 °C in terms of performance (peak power density, mW·cm^−2^) *vs.* membrane selectivity, a steep increase of performance with selectivity is observed for low values of selectivity, whereas the trend is less steep when the selectivity of the membrane further increases (Figure 4). In principle, the performance should approach a plateau for very high selectivity values, e.g., for membranes with low area0specific resistance and very low crossover. In the investigated range, ionic conductivity appears sufficiently high and membrane thickness reasonably low for DMFC applications, whereas methanol crossover should be further decreased. The observed trend would suggest an increase of performance with selectivity according to a square root function. In the presence of very small methanol permeability, the anode characteristics should dominate the cell performance, providing that the membrane is characterized by suitable area-specific resistance. Thus, the membrane selectivity (defined as indicated above) appears to be a key parameter to guide the development of DMFC membranes for portable and APU applications. In particular, the area-specific resistance, R_s_ (high frequency intercepts in the Nyquist plot) includes the contributions of both membrane thickness and proton conductivity. Thus, the selectivity parameter here defined resumes the contribution of the most important membrane features: crossover, conductivity, and thickness.

It should be pointed out that this analysis covers different families of membranes such as low-EW PFSA (Nafion^®^), high-EW PFSA, sPEEK, composite PFSA membranes with inorganic fillers, cross-linked membranes, *etc*. Since the interface characteristics also play a relevant role, some data scattering is observed for this relationship. The best interface properties should occur for Nafion 115 membrane since the catalytic layers contain an ionomer of the same chemical composition. The Nafion ionomer dispersion was kept constant in the various MEAs.

At low temperature conditions (60 °C), the electrode–electrolyte interface probably plays a minor role provided that MEAs are assembled using the same procedure.

An appropriate regression of the performance data recorded at 60 °C gives a square root relationship between power density and selectivity, as reported below:

P.D. = k s^0.5^. (3)

At 60 °C, P.D. ≈ 260 s^0.5^.

Thus the power density can be estimated, for this series of MEAs, from the square root function of the selectivity and a multiplication factor being P.D. ≡ mW·cm^−2^, s ≡ Ohm^−1^·mA^−1^ and the multiplication factor: k ≡ V·mA^1.5^·Ohm^0.5^·cm^−2^.

**Figure 5:**
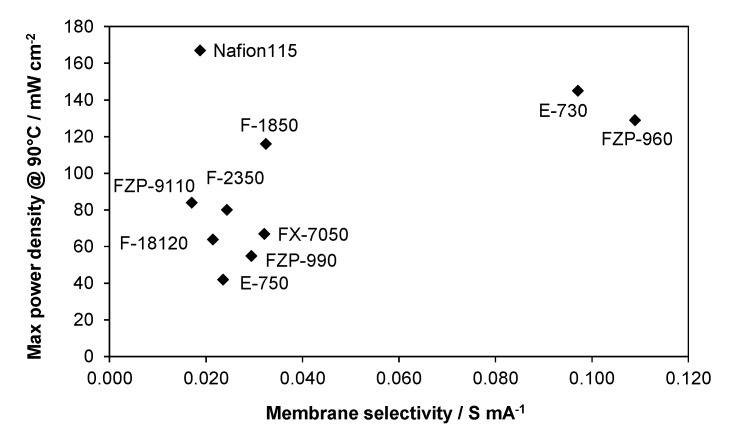
Performance *vs.* selectivity for MEAs equipped with different membranes at 90 °C (2 M MeOH).

Less evident is the trend at 90 °C (Figure 5). In this case, there are just two groups of membranes: one having low selectivity and showing very low or very high power density; and a second group with just two membranes having high selectivity and high power density. The square root function cannot be used to fit the data at 90 °C with such large scatter. It is reasonable to consider that, since at 90 °C the effect of cathode poisoning by methanol crossover is less relevant (the selectivity parameter includes the crossover effect), the effect of the quality of the electrode–electrolyte interface plays a major role in determining the performance [14,27]. This is quite clear for Nafion^®^ since the ionomer dispersion used in the electrodes was of the same composition as in the membrane, whereas, for the other membranes, there may be less compatibility with the Nafion^®^ ionomer in the electrodes.

**Figure 6:**
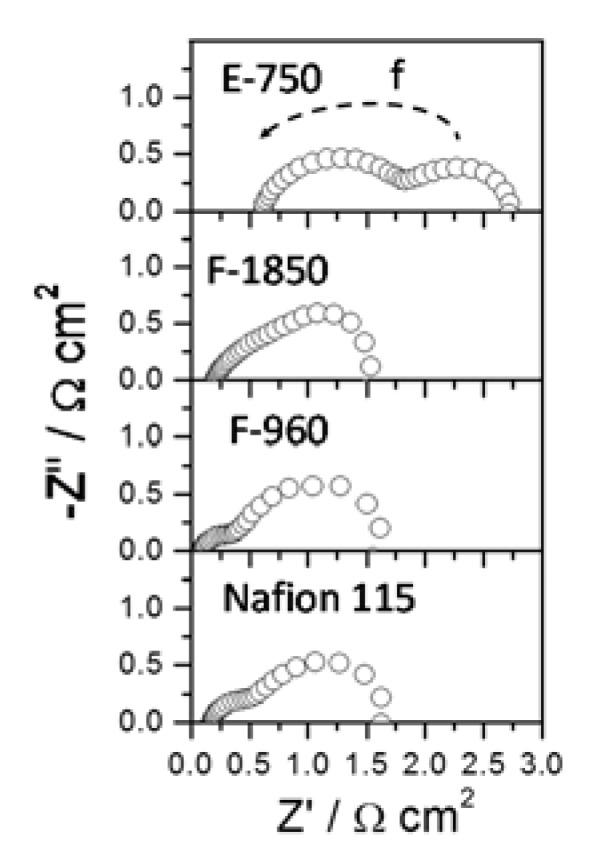
Ac-impedance plots for selected MEAs equipped with different membranes at 60 °C (2 M MeOH, 75 mA·cm^−2^).

Ac-impedance spectra for selected membranes, recorded in the galvanostatic mode *i.e.*, at a current density of 75 mA·cm^−2^, are shown in Figure 6. This current density corresponds to the region where activation phenomena and methanol crossover play a relevant role. Two overlapping semi-circles are evident. The ac-impedance profile of the DMFC cell is similar to that generally reported in the literature [28,29]. In previous studies, the semicircle at low frequencies was in good part associated to the cathode poisoning by methanol crossover [28,29]. Thus, it can be underlined that under such conditions the total impedance is significantly affected by the crossover of methanol. At higher current densities, the poisoning effect of the crossover decreases whereas series resistance (high frequency intercept on the x-axis), mainly associated with the membrane conductivity and thickness, remains almost constant. However, the contribution of the series resistance to the total impedance increases as the current density increases because of the decrease of the polarization resistance (the difference between the low and high frequency intercept on the x-axis) [28]. Ac-impedance analysis indicates that the effect of cathode poisoning by methanol crossover is more significant than the series resistance in a relevant portion of the polarization curve corresponding to high voltage efficiency for most of the membrane systems investigated here. The situation is different for membranes with low conductivity (e.g., E-750), especially at high current densities. The overall results clearly show that the membrane selectivity is one of the main parameters determining the peak power density of DMFCs in the low temperature range (60 °C).

## 3. Experimental Section

**Figure 7:**
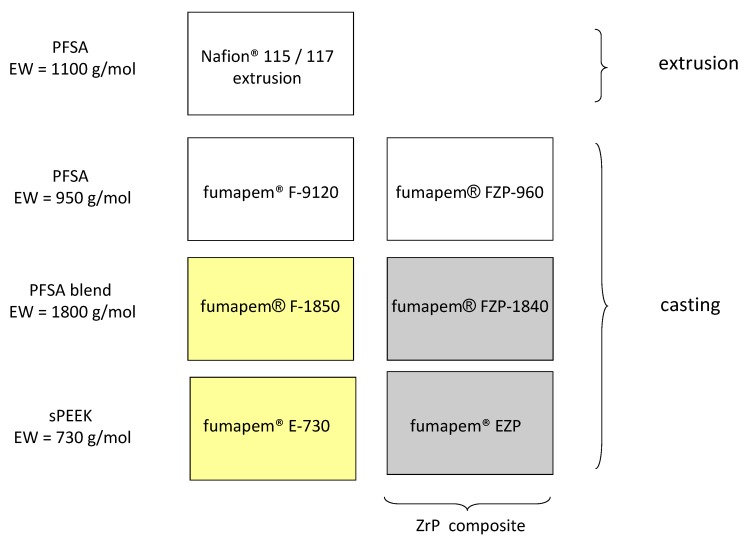
Fumatech membranes varying in terms of equivalent weight and preparation procedures for DMFC applications.

Long side chain (Fumion^®^) PFSA blends with 1800 and 2300 equivalent weight (e.g., fumapem^®^ F-1850, F-18120, and F-2350), hydrocarbon membranes based on sPEEK (e.g., fumapem^®^ E-730 and E-750), composite membranes based on zirconium phosphate (ZrP = Zr(HPO_4_)_2_·H_2_O) and perfluorosulfonic acid polymer (e.g., fumapem^®^ FZP 960 and FZP 990), and cross-linked PFSA membranes (e.g., FX-7050) were prepared at Fumatech using procedures reported elsewhere [20,21,22,23,30,31]. The main characteristics of these Fumatech membranes in terms of composition, equivalent weight, and preparation methods are reported in Figure 7.

All the membranes were prepared by solvent casting or extrusion.

Solvent uptake, proton conductivity, and other relevant membrane properties were determined. *Ex situ* conductivity was measured in-plane using a four-electrode setup with the membrane in demineralized water at 25 °C. The cell was connected to an ac-impedance spectrometer. Dimensional swelling was determined from the increase in membrane dimension (x, y, z directions) on immersion (4 h) in water and methanol. For IEC determination, a treatment of the membrane sample with 0.1 M NaCl at room temperature was carried out followed by 12 h titration of solution with 0.1 M NaOH. Solvent uptake of the various membrane samples was carried out by immersing the sample in the solvent at 25 °C for 1 h followed by removal of solvent from surface by tissue paper and weighting the dry sample in a vacuum over P_2_O_5_ at 50 °C to get a measure of the dry weight.

The developed membranes were integrated in membrane–electrode assemblies and investigated in a direct methanol fuel cell in terms of performance and methanol crossover. The same electrodes were used in all experiments. The electrodes consisted of catalytic layers based on 15 wt % Nafion ionomer (Ion Power, 5 wt % solution, 1100 EW) and 85 wt % catalyst, and commercial backing layers (E-TEK). A Pt−Ru/C catalyst (1:1 atomic ratio) was used as the anode catalyst; a Pt/C catalyst was used at the cathode for all MEAs. The same catalyst loading was used for all MEAs. The loadings were 2 mg (Pt + Ru) cm^−2^ for the anode and 1 mg·cm^−2^ Pt for the cathode.

Regarding MEA fabrication, the type of lamination procedure is generally strongly dependent on the glass transition temperature of the membrane; thus, inappropriate hot pressing procedures may affect the comparison of membrane characteristics. Lamination at high temperature and pressure may also affect the crossover characteristics of the membrane by causing membrane thinning and/or penetration of the catalytic layer inside the membrane during compression.

To assess the different membranes and to compare their behavior to Nafion^®^ in a context where their properties are not affected by the lamination procedure, MEAs have been assembled *in situ*. The compression was kept constant for all the MEAs at 15 kg·cm^−2^. No thermal treatment was carried out during the MEA assembling. The recorded performance may be thus lower under these assembling conditions, but the aim of this analysis was essentially to provide a comparison of the membrane characteristics in MEAs. These polymer electrolytes have been investigated in direct methanol fuel cells both at low (60 °C) and high (90 °C) temperatures and compared to the benchmark Nafion^®^ 115 membrane.

The MEAs were tested in a 5-cm^2^ single cell using a DMFC test station (Fuel Cell Technologies). The cell was also connected to an Autolab PGSTAT 302 Potentiostat/Galvanostat (Metrohm) equipped with a FRA impedance module. For polarization curves, a 2 M methanol solution was fed at the anode with a flow rate of 3 mL·min^−1^, whereas the oxidant was fed at the cathode under atmospheric pressure (100 mL·min^−1^). Area-specific resistance was measured by electrochemical impedance spectroscopy (EIS) from the high frequency intercept on the real axis of the Nyquist plot. Ac-impedance spectra were recorded in the galvanostatic mode at 75 mA·cm^−2^ at 60 and 90 °C.

Methanol crossover for different concentrations of 1, 2, or 5 M (3 mL·min^−1^) and temperatures of 30°, 60°, or 90 °C, was determined by chromatographic analysis and electrochemical methods. The exhaust gas at the cathode side of a 5 cm^2^ DMFC single cell operating under open circuit voltage (OCV) was analyzed by using an online micro-gas chromatograph (Varian). The CO_2_ produced by the oxidation of methanol, permeated to the cathode, was determined. Methanol crossover was also determined electrochemically with the DMFC operating in the driven mode and using CH_3_OH (3 mL·min^−1^) at the anode side and He (100 cm^3^·min^−1^) at the cathode. The methanol permeating the MEA was oxidized at cathode (Pt catalyst) generating a positive current, which reached a plateau when all methanol was converted to CO_2_ under steady state conditions [26]. The crossover measurements were carried out by using the linear sweep voltammetry (LSV) mode with a voltage scan rate of 2 mVs^−1^ and in the voltage range from 0 to 0.95 V. A Metrohm Autolab instrument was used for performing the linear sweep voltammetry.

## 4. Conclusions

FuMA-Tech developed a new generation of fluorinated membranes for DMFC application. Several of these systems had ion exchange capacities of 0.4 and 0.5 meq/g, which corresponded formally to equivalent weights of 1800 and 2300 g/mol, respectively. These values were significantly different from those of standard PFSA membranes e.g., Nafion^®^ 115, of equivalent weight 1100 g/mol and IEC 0.91 meq/g. These blend membranes were formulated to limit methanol crossover, while the cast membrane thickness of 30–50 µm allowed us to reduce the membrane area resistance compared to Nafion^®^ 115, with a thickness of 125 µm, and to decrease material cost. Sulfonated polyetheretherketone membranes and composite membranes with zirconium phosphate were also developed at FuMA-Tech.

A square root-like relationship between DMFC power density and membrane selectivity (reciprocal of the product between the area-specific resistance and the crossover) was observed at 60 °C. This relationship can provide a guideline to predict DMFC performance from basic membrane characteristics in the presence of similar catalyst-loading, mechanical, and interfacial properties.

As discussed above, the selectivity is related to the intrinsic membrane properties. Thus, determination of membrane selectivity does not necessarily require to carry tests in DMFCs. Selectivity can be determined from conductivity, thickness, and methanol permeation characteristics. According to our evidence, membrane selectivity can give an indication of DMFC performance at low temperatures, provided that the electrode properties are known. This is a good opportunity for membrane developers who do not have an experimental DMFC setup or are not involved in electrochemistry to develop new polymeric membranes for methanol fuel cells and get an estimation of the performance achievable in DMFC under conditions that are relevant for portable applications.

The achievement of proton conductivity for the developed membranes in the range of 50 mS/cm, in addition to having satisfactory properties with respect to methanol permeation, hydrolytic, and mechanical degradation, provided a set of polymer electrolyte materials with enhanced characteristics for methanol fuel cells. This enabled an increase in DMFC performance (power density) with respect to the reference state-of-the-art Nafion^®^ membrane at 60 °C.

The FuMA-Tech E-730 s-PEEK membrane provided similar or higher power density than Nafion^®^ in the 60–90 °C range and lower crossover despite the much lower thickness resulting in a better fuel utilization and higher energy density, and lower polymer cost. Among the most promising membranes, sulfonated polyetheretherketone E-730 showed several advantages compared to Nafion^®^ in terms of power density (about 77 mW·cm^−2^ as compared to 64 mW·cm^−2^ at 60 °C on a low catalyst loading basis), lower methanol (2M) crossover (47 mA·cm^−2^ for E-730 *vs.* 120 mA·cm^−2^ equivalent current density for Nafion^®^ 115 at 60 °C) and suitable area-specific resistance (0.15 Ohm cm^2^ for E-730 *vs.* 0.22 Ohm cm^2^ for Nafion^®^ 115). The significantly lower polymer cost for E-730 compared to Nafion^®^ also provides interesting perspectives for DMFC application. E-730 consists of a cheap hydrocarbon membrane (PEEK) and no fluorine chemistry is involved. This is obtained by a cost-effective process instead of the perfluorinated Nafion^®^. The lower cost is also associated with the much lower amount of polymer used in the cell due to the lower thickness. Another Fumatech membrane, a perfluorinated-type, F-1850, was also promising and potentially can cover a wide range of operating temperatures. This membrane is also reasonably cheap even if based on PFSA; it already provides a cost advantage over non-blended PFSA (Nafion^®^, *etc.*). In particular, the F1850 provided a proton conductivity of 46 mS·cm^−1^ at 60 °C. ZrP-PFSA composite membranes showed conductivities between 40 and 70 mS·cm^−1^ but large methanol crossover. From the above results, it is deduced that the developed cost-effective polymer electrolytes can provide a performance comparable to or better than Nafion^®^, especially at low temperatures, and better fuel utilization in DMFCs.
